# Supplementary material for: Altered corticospinal excitability of scapular muscles in individuals with shoulder impingement syndrome
Source: PLoS One. 2022 May 16;17(5):e0268533. doi: 10.1371/journal.pone.0268533 (PMC9109916; doi:10.1371/journal.pone.0268533)
Supplement: S1 Data — (PDF) [file pone.0268533.s001.pdf]

Group	UT_AMT	LT_AMT	SA_AMT	UT_CSP	LT_CSP	SA_CSP	UT_Lat	LT_Lat	SA_Lat
1	0.43	0.46	0.52	27.02	30.12	52.98	8	9.84	9.12
1	0.55	0.48	0.48	93.24	31	95.04	11.08	14.32	12.72
1	0.42	0.46	0.49	59.92	48.36	63.48	7.92	12.52	10.12
1	0.47	0.46	0.56	61.4	36.4	87.64	8.88	10.72	12.52
1	0.54	0.6	0.63	95.84	44.2	52	7.44	10.36	10.84
1	0.45	0.44	0.47	40.68	47.64	62.52	7.16	9.08	13.04
1	0.48	0.48	0.5	39.16	36.12	30.36	7.36	9.72	12.2
1	0.42	0.54	0.48	56.04	60.68	53.92	7.6	11.28	16.28
1	0.52	0.6	0.5	56.12	48.88	53.24	5.96	12.6	14.28
1	0.5	0.58	0.58	66.68	38.32	67.28	8.4	9.92	14.24
1	0.47	0.6	0.58	47.6	54.08	71.6	9.28	11.16	16.8
1	0.49	0.61	0.59	106.4	83.8	99.56	8.84	9.92	13.52
1	0.55	0.52	0.54	46.16	27.62	31.5	9.58	12.86	12.4
1	0.62	0.66	0.65	43.96	45.8	81.88	8.92	11.24	9.88
2	0.5	0.66	0.58	34.84	52.8	42.32	8.52	8.76	15.16
2	0.52	0.55	0.58	88.04	92	76.76	10.84	12.2	12.96
2	0.53	0.62	0.65	41	48.8	47.88	8.52	10.44	11.88
2	0.5	0.5	0.62	52.16	61.68	112.44	8.6	13.48	13.6
2	0.61	0.7	0.63	38.72	51.48	50.44	9.56	11.84	15.12
2	0.58	0.66	0.58	44.72	69.12	63.68	7.44	8.48	8.84
2	0.66	0.67	0.56	145	101.72	71.56	7.56	8.84	10.2
2	0.46	0.57	0.54	60.76	58.8	99.64	8.68	11.12	3.2
2	0.5	0.53	0.62	43.04	45.52	62.68	6.92	9.28	10.12
2	0.52	0.66	0.61	40.92	57.12	94.08	9.2	7.96	17.36
2	0.55	0.6	0.59	109.64	60.52	115.88	8.68	11.6	11.08
2	0.5	0.52	0.51	34.12	42.08	152.32	14.08	8.44	14.76
2	0.56	0.6	0.62	37.84	24.92	66.48	8.28	10.96	9.12
2	0.48	0.54	0.58	100.72	104.68	119	9.2	12.52	13.56

H_mean	0.493571	0.535	0.540714	60.01571	45.21571	64.5	8.315714	11.11	12.71143
H_SD	0.057727	0.072404	0.058502	23.39875	14.63559	21.20367	1.254804	1.489342	2.262371
P_mean	0.533571	0.598571	0.590714	62.25143	62.23143	83.94	9.005714	10.42286	11.92571
P_SD	0.054152	0.064672	0.037716	34.66037	22.86823	32.36056	1.748906	1.784978	3.565436

1: healthy group
2: Patient group

UT: upper trapezius
LT: lower trapezius
SA: serratus anterior

UT_MEP	LT_MEP	SA_MEP	UT_COGx	UT_COGy	LT_COGx	LT_COGy	SA_COGx	SA_COGy	UT_Area
2.58	0.31	0.542	2.1	2.13	2.75	1.3	2.011	2.094	15.98
0.33	0.55	0.2736	2.64	2.06	3.45	2.66	3.325	3.04	22.87
0.8	0.27	0.188	3.29	2.93	3.79	5.55	4.295	5.454	28.95
3	0.56	0.3216	3.9	5.41	4.03	5.88	4.367	5.445	26.34
0.63	0.61	0.3402	2.14	2.6	1.86	2.62	2.183	2.76	22.37
0.3636	1.3834	0.7012	2.75	2.6	2.79	2.49	3.075	2.312	22.88
0.6972	0.5636	0.2028	4.28	4.46	3.81	5.99	3.842	5.51	32.88
1.06	0.87	0.8064	4.42	2.18	4.21	2.31	4.793	2.387	18
0.65	0.77	0.4194	2.82	1.65	3.07	1.54	3.178	1.156	18.89
0.65	0.83	0.3578	4.02	2.6	4.09	2.38	4.456	3.1	31.12
1.06	0.58	0.3214	3.44	2.59	2.94	2.34	3.137	2.504	29.62
3.11	1.95	0.575	4.45	-0.488	4.4	-1.059	4.568	-0.654	30.85
0.382	0.4898	0.5394	3.25	5.59	3.95	5.4	3.811	6.003	23.22
1.4686	1.81	1.4492	4.295	2.26	4.37	2.62	4.295	2.511	25.39
0.38	0.75	0.2906	3.29	1.3	2.42	1.87	3.095	1.923	16.28
1.66	1.33	0.4686	2.65	2.23	3	2.19	2.784	2.153	16.39
1.02	1.81	0.3666	3.24	2.11	2.74	2.01	3.132	2.141	25.08
0.85	1.26	0.3554	3.13	1.38	3.22	1.47	3.507	1.817	15.43
1.33	0.54	0.2732	4.04	2.83	3.92	3.67	4.5	3.033	25.04
2.08	1.09	0.6406	3.61	1.31	2.93	0.92	3.387	1.298	17.63
1.34	0.55	0.7544	3.7	4.18	3.39	4.21	4.345	4.768	27.9
1.23	1.1808	1.76	3.21	1.56	2.89	1.81	3.33	1.7	21.41
0.57	1.12	0.3792	3.65	2.88	3.08	2.7	4.403	2.959	26.11
1.52	0.51	0.5452	3.37	1.87	3.2	1.23	3.55	1.538	17.71
2.88	0.99	0.76	3.19	2.38	3.49	2.6	3.02	2.74	28.36
1.4192	1.7158	0.8588	3.16	0.39	3.22	-0.142	3.115	0.555	43.91
1.4844	0.4206	0.4522	2.11	-0.032	2.504	0.695	2.467	1.225	13.29
1.95	0.78	0.5762	2.9	1.97	3.11	1.87	2.482	1.935	19.8

1.198671	0.824771	0.502714	3.413929	2.755143	3.536429	3.0015	3.666857	3.115857	24.95429
0.975552	0.523213	0.327824	0.832065	1.551936	0.750452	2.018275	0.875494	1.88326	5.258296
1.408114	1.003371	0.605786	3.232143	1.882714	3.079571	1.935929	3.3655	2.1275	22.45286
0.635941	0.438608	0.379419	0.475365	1.056684	0.389504	1.140277	0.657181	1.020574	7.91282

LT_Area	SA_Area	Age	Weight	Height	BMI
20.67	21.005	25	52	1.58	20.83
21.28	30.855	25	46	1.53	19.65056
27.83	41.181	25	64	1.66	23.22543
28.11	41.991	24	70	1.75	22.85714
22.28	14.863	24	72	1.68	25.5102
16.58	21.569	25	47	1.55	19.56296
15.68	27.566	26	50	1.55	20.81166
23.17	23.464	26	47	1.68	16.65249
22.76	23.143	30	44	1.58	17.62538
30.49	37.216	23	80	1.78	25.24934
26.07	22.044	24	87	1.81	26.55597
37.077	40.562	20	64	1.7	22.14533
11.03	8.456	24	63	1.75	20.57143
16.63	23.045	24	65	1.72	21.97134
19.42	13.191	24	58	1.64	21.56455
23.59	21.207	23	54	1.58	21.63115
21.97	26.568	28	60	1.66	21.77384
20.55	25.675	24	66	1.82	19.92513
10.56	34.972	23	80	1.83	23.88844
11.62	19.245	22	48	1.54	20.2395
19.78	28.989	25	60	1.67	21.51386
24.21	29.13	22	59	1.74	19.48738
20.75	24.978	21	53	1.6	22.09317
15.85	20.806	21	70	1.78	29.21841
23.41	30.12	29	57	1.67	20.43817
36.24	35.931	24	75	1.78	23.67125
6.42	13.732	22	55	1.68	19.48696
21.06	18.178	23	100	1.85	20.70313
22.83264	26.92571	24.64286	60.78571	1.665714	21.65852
6.795839	10.20345	2.134232	13.54297	0.093044	2.885992
19.67357	24.48014	23.64286	63.92857	1.702857	21.83107
7.188224	7.09983	2.373156	13.62387	0.098248	2.524362
