# Supplementary material for: Altered corticospinal excitability of scapular muscles in individuals with shoulder impingement syndrome
Source: PLoS One. 2022 May 16;17(5):e0268533. doi: 10.1371/journal.pone.0268533 (PMC9109916; doi:10.1371/journal.pone.0268533)
Supplement: Attachment — Submitted filename: Response to Reviewers.docx [file pone.0268533.s002.docx]

Reviewer #1

General comments:

1. Yi-Fen Shih et al. compared TMS variables of three scapular muscles (serratus anterio, superior and inferior trapezius) in participants with and without impingement syndrome. They observed significant differences between multiple variables (e.g. AMT, CoGy). I have several methodological concerns that could make the results un-interpretable. TMS was tested during shoulder scaption and EMG was not controlled during this movement. All the differences between groups could come from the absence of EMG control. Indeed, higher EMG activity in one group could result in differences in AMT or CoG. Authors need at least to provide EMG activity of the three muscles during TMS testing.

Ans: Thank you for pointing out this issue. Because this investigation was conducted between 2014 and 2015 when using EMG activity to normalize the TMS data was less popular, we did not collect the EMG data when measuring TMS. We added discussion regarding the lack of EMG control in the limitations: “Our TMS data was recorded and analyzed without EMG activity control. This might impact some of the TMS variables such as AMT and CoG.” (P16 Ln271-274)

2. The TMS session was really ambitious. Testing hostpot, AMT and mapping of three different muscles would certainly take 3-4 hours. Did participants report pain and fatigue during testing?

Ans: Thank you for raising this question. The whole testing session took around 3-4 hours and most of the participants reported not much pain, but fatigue. This information is added in the Results (P10, Line 169-170) and discussed in the limitations. (P17 Ln277-283)

3. The location of MEG electrodes are not specified. Considering that the lower trapezius and serratus anterior are difficult to locate and test using EMG, more details are need in the methods on how the electrodes were placed and how authors did confirm positioning over these muscles. Also, a lot of cross-talks could come from adjacent muscles.

Ans: Thank you for reviewer’s suggestion. The details of the EMG electrode locations are added in the 2.2 Corticospinal excitability assessment, Procedure (P6, Ln101-107).

4. Data are not all available in a public repository. Only means and a measure of dispersion are available in the Figures.

Ans: Raw data is now available in Supplement 1.

Specific comments:

Introduction

5. Ln 40-41: I don’t think that we can assure that scapular dyskinesis is the leading factor of SIS. I would be more appropriate to indicate that this is one of the factor.

Ans: Thank you for reviewer’s suggestion. The sentence is revised accordingly:” Multiple factors contribute to SIS and one of them is scapular dyskinesis (P3 Ln40).”

6. Ln 53: Typo with reference (7)

Ans: Thank you for reviewer’s reminder. This typo has been corrected. (P5 Ln 51)

7. Ln 55: I don’t think we can use the term muscle kinetics that refer more to the biochemistry of muscle contraction rather than muscle activation. I think motor control may be a more appropriate term. Also the sentence is a bit trivial, it is clear that scapular dyskinesis suggest alteration of scapular motor patterns. Dyskinesis means modification of movement.

Ans: Thank you for reviewer’s comment. We have deleted the redundant sentence.

8. Ln 63: Typo: recently should be recent

Ans: Thank you for reviewer’s reminder. This typo is corrected. (P4 Ln59)

9. Ln 64-66: Authors should use the term lower rather than decrease since it is cross-sectional studies. Decrease/increase infers that pain induced this effect, but we cannot conclude this from cross-sectional studies. Other studies also showed lower in intracortical inhibition level of trunk muscles in chronic low back pain compared to pain-free controls 12.

Ans: Thank you for reviewer’s valuable suggestion. The description is revised accordingly, using “lower” and “higher” instead of decreases or increases. (P4 Ln63-64)

10. Ln 70: What do authors mean by corticospinal inhibition? Do they mean intracortical inhibition? Typo: motor representation rather than presentation.

Ans: Thank you for reviewer’s reminder. We changed the “corticospinal inhibition” to “intracortical inhibition”, and the typo is corrected. (P4 Ln69-70)

Material and methods

11. Ln 78: How the effect size was calculated?

Ans: This effect size was calculated based on the AMT of the upper trapezius from our pilot study. This information is added. (P5 Ln 78)

12. Ln 95-98: It is important to provide more details about the exact locations of the EMG electrodes for each muscle tested. How do authors ensure that the EMG electrodes recorded muscle activity from the muscles targeted? Specifically, considering that the trapezius inferior and serratus anterior may be difficult to locate and palpate, what techniques did authors use to ensure proprer electrode placement?

Ans: Thank you for reviewer’s suggestion. The details of the EMG electrode locations are added in the 2.2 Corticospinal excitability assessment, Procedure (P6, Ln101-107).

13. Ln 109: Why using a 90-degree scaption during TMS testing?

Ans: Thank you for the question. We found it difficult to elicit TMS responses in the resting position, and the 90-degree scaption is a functional position for the shoulder complex at which our target muscles would generate about 10-15% muscle activation for TMS assessment. (P8, Ln119-122)

14. Ln 117: type: grid rather than grip

Ans: Thank you. This typo is corrected. (P9, Ln126)

15. Ln 113-121: Did authors tested motor threshold before finding the hotspot? The order in which this is presented let is supposed this. If so, this is problematic.

Ans: Thank you for raising this issue. We started with 70-80% of the maximum stimulator output (MSO) to find the hot spot first, and then gradually decreased the intensity to find the lowest stimulator intensity as the active motor threshold (AMT). The sentence is corrected to clarify the confusion. (P9, Ln126-131)

16. Ln 113-121: It seems like hostpsot and AMT of each muscle was measured independanlty. This takes usually a lot of time. Can authors provide how long it took to measure hotspot and AMT of each individual muscle?

Ans: Thank you for raising this concern. We did measure the hotspot and AMT independently for each muscle. Because the hotspots and AMTs of these three muscle are not far from each other, it was not too troublesome to do it one by one. It generally took us about an hour to do all three.

17. Ln 127-129: Why did only the 5 fist MEP amplitude were averaged? This is unconventional.

Ans: Thank you for the question. We average the five highest EMP out of 10 trials, not the first five. The sentence is corrected. (P9, Ln139-140)

18. Ln 135: Please use references when potential mechanisms for TMS variables are stated (e.g. 3).

Ans: Thank you for the suggestion. The reference is added. (P9 Ln148)

19. Ln 139-141: Why a 11 µV MEP amplitude is used as cut-off? Why not using the same cut-off as for the AMT?

Ans: Thank you for raising the question. This criterion came from Wassermann et al. (1992)’s protocol, and the reference is added. (P9 Ln 152)

20. The total duration of the session should be mentioned. This is a really long and ambitous session with three muscles tested for hotspot, AMT and mapping. How did the participants cope with such a long session? Did they report pain or fatigue? If so, how fatigue and pain may affect the results?

Ans: Thank you for the suggestion. The whole testing session took around 3-4 hours and most of the participants reported not much pain, but fatigue. This information is added in the Results (P10, Line 168--170) and discussed in the limitations. (P17 Ln278-284)

Results:

21. It is critical to control the level of EMG that was present during TMS. Differences between groups may be driven by an increase EMG activity during the scaption movement. For exmaple, for mapping, a previous study showed that CoG was different at rest vs. in activity. Was there differences in EMG activity for the three muscles during TMS?

Ans: Thank you for raising the concern. We realized the importance of EMG activity control for the TMS data. Because this investigation was conducted between 2014 and 2015 when using EMG activity to normalize the TMS data was less popular, we did not collect the EMG data when measuring TMS. We added discussion regarding the lack of EMG control in the limitations: “Our TMS data was recorded and analyzed without EMG activity control. This might impact some of the TMS variables such as AMT and CoG.” (P17 Ln273-274)

Reviewer #2:

In current manuscript authors have assess and compare the corticospinal excitability, corticospinal inhibition and motor presentation of the upper and lower trapezius, and the serratus anterior in participants with and without SIS.In general manuscript is written well but for better presentation of the manuscript i would like authors to comment so following points

Abstract:

1. Page 3, Ln 23:Mention sampling technique used for recruitment of participants

Ans: Thank you for reviewer’s comment. We used convenient sampling (leaflets) to recruit participants. The information is added. (Abstract, Ln24)

2. Page 3, Ln 28: A sentence on how the data was analyzed will be useful

Ans: Thank you for reviewer’s suggestion. We used Mann-Whitney U tests to analyze the data. The information is added. (Abstract, Ln28)

3. Page 3, Ln 29-33: Provide p-values for the results

Ans: Thank you for reviewer’s suggestion. All p-values are added. (Abstract, Ln30-34)

4. Page 3, Ln 35: Shift “in” motor presentation

Ans: Thank you for reviewer’s reminder. The “in” is added. (Abstract, Ln35)

Introduction:

5. Page 5, Ln 64: The term “individuals with musculoskeletal pain” is very broad. Please be more specific about the population in which the changes were reported. Were these studies done on people with shoulder pain or any musculoskeletal pain at any site?

Ans: Thank you for reviewer’s comment. These included low back pain, chronic ankle instability, rotator cuff tendinopathy, and anterior cruciate ligament injury. The information is added. (P4, Ln60-61)

6. Page 5, Ln 64: Unclear what “changes in the center of gravity of a cortical map” means..

Ans: Thank you for reviewer’s reminder. We rephrased the sentence to clarify the confusion as” changes in the location of center of gravity…” (P4, Ln62)

Methods:

7. Page 6, Ln 77: Please provide details on participant recruitment i.e., how and from where were the participants recruited for the study?

Ans: Thank you for reviewer’s reminder. We used convenient sampling (leaflets) to recruit participants from the local community. The information is added. (P5, Ln77)

8. Page 6, Ln 78: Please provide more details about the inclusion criteria of participants. What was the age group that was targeted? The criteria about age group appears for the first time in the second last paragraph of the discussion section. This is needs to be mentioned in the methods section. Was pain intensity, pain duration, cause of impingement: traumatic or non-traumatic and bilateral or unilateral involvement used to screen eligible participants?

Ans: Thank you for reviewer’s suggestion. We add detailed inclusion criteria in the “participant” section. (P5 Ln79-84)

Page 6,Ln 80-83:Please provide references for the cluster of tests used for the inclusion of patients.

Ans: Thank you for the suggestion. The reference 16 is added.

Magee DJ, Manske RC. Orthopedic Physical Assessment: Elsevier - Health Sciences Division; 2021.

9. Page 6, Ln 82: Add “examined” before “by a licensed…” and replace “physical therapy” with “physical therapist”.

Ans: Thank you for reviewer’s suggestion. The sentence is revised accordingly. (P5 Ln83-84)

10. Page 8, Ln 108, 109: Was the arm held in scaption decided based on side affected or contralateral to testing hemisphere?

Ans: Thank you for raising the concern. The testing arm was the affected side. The sentence is revised to clarify the confusion. (P7 Ln117)

11. Page 10, Ln 150: Please provide the justification for using a non-parametric Mann-Whitney U test. Please provide information on whether the data was normally distributed.

Ans: Thank you for raising the concern. A non-parametric test was used because of the small sample size and some our data were not normally distributed as shown by histograms. This information is added. (P10, Ln162-163)

Results:

12. Page 11 Ln 155: Please change the tense to past tense in Ln 155

Ans: Thank you. The typo is corrected accordingly. (P10, Ln168)

13. Table 1 shows that participants pain intensity was collected using VAS scale, but this has not been mentioned in the methods section. Please provide more details about participant characteristics such as side of affected shoulder, pain duration (acute or chronic condition), does the VAS pain rating represent pain at rest or pain during movement, pain on the day of testing or average pain in past week/month.

Ans: Thank you for the suggestion. The information is added in the Methods (chronic pain for at least 3 months, the most pain experienced during movement etc.) (P5, Ln80-90) and Table 1.

Discussion:

14. Page 12 Line 170-172: Recheck sentence structure and rephrase. Include which side corticospinal excitability is increased or decreased i.e., ipsi- or contralateral. Please include the clinical implication of the findings in the first paragraph of the discussion section.

Ans: Thank you for reviewer’s suggestion. We revised the paragraph as suggested “Our results showed that the participants with SIS had lower contralateral corticospinal excitability (higher AMT) in the lower trapezius and serratus anterior, and higher contralateral intracortical inhibition (longer CSP) in the lower trapezius, and changes in contralateral cortical representation of the upper trapezius and serratus anterior. These findings reflected that SIS was associated with reorganization of corticospinal system of the scapular muscles, which should be concerned when managing people with SIS.” (P12 Ln186-191)

15. Page 15, 234: Replace “studies” with “study”

Ans: Thank you. The “studies” is replaced by the “study”. (P16 Ln271)

16. Page 17, Line 254-269: Please include some suggestions on how the limitation listed for the current study can be improved in future research.

Ans: Thank you for reviewer’s suggestion. We add some information regarding how to improve the study methods in the “limitation”. (highlighted, P16 Ln 271-294)

17. In table two on line 387 please mention the test for the P valuse as done in line 380 for table 1.

Ans: Thank you for the suggestion. The information is added in Table 2.

18. Please improve the figure 2 ,3A and 3B quality as currently they arr blurry.

Ans: Thank you for reviewer’s suggestion. We re-do the figures with better resolutions. (Figure 2 and 3)
