# Supplementary material for: Altered corticospinal excitability of scapular muscles in individuals with shoulder impingement syndrome
Source: PLoS One. 2022 May 16;17(5):e0268533. doi: 10.1371/journal.pone.0268533 (PMC9109916; doi:10.1371/journal.pone.0268533)
Supplement: Attachment — Submitted filename: Response to Reviewers_R2_0221.docx [file pone.0268533.s003.docx]

Reviewer #1

General comments:

1. I thank authors for considering my comments. However, there are some authors’ responses for which I am not convinced and other elements I would like them to address. About EMG control, in 2014-2015, it was well known that EMG background amplitude (representing the net motoneuronal excitability) was critical to control in TMS research considering the MEP amplitude represent the excitability at both cortical and spinal levels. Authors may control for EMG by measuring pre-stimulus EMG background to determine if it was similar between groups for each muscle tested. For example, if participants of the SIS group recruit more the lower trapezius during the task, it may explain the longer duration of the CSP or the difference in AMT. Further analyses are required to ensure that the results are not due to different patterns of recruitment in the different groups.

Ans: Thank you for reviewer’s suggestion. Unfortunately the raw EMG set has been deleted, we were unable to retrieve it for further analysis. Some of the participants (6 in each group) were tested in the preliminary study for EMG activation of the three target muscles during scaption, and we found no group difference in EMG activities of the upper and lower trapezius and serratus anterior (p values ranging from 0.26 to 0.99). This information was added in the final paragraph of the Discussion. (Line 278-284)

2. I encourage authors to review the literature and report TMS studies comparing patients with and without musculoskeletal pain for cortical and corticospinal control. For example, differences in AMT 1 and in intracortical inhibition 2. Considering that these variables were not reported in TMS studies of shoulder muscles, it may help for the discussion of the results.

Ans: Thank you for reviewer’s valuable suggestion. References are added as suggested. (P4 Ln60-69)

3. About the use of the 5 highest MEP out of 10, why did the authors used these methods? Could they use references and discuss how it could impact on the results?

Ans: Thank you for pointing out the question. This method was chosen for better measurement reliability based on the Cavaleri et al.’s review, and this information is added. (P8 Ln142-145)

4. Ln. 64-67: In this sentence, it should be clear that the difference was with the non-painful side, and not with a control group.

Ans: Thank you for reviewer’s suggested. The sentence is revised accordingly to avoid the confusion. (P4 Ln65)

5. About sample size calculation: what was the alpha and beta levels used?

Ans: Thank you for reviewer’s reminder. The information is added (effect size: 0.724, alpha level: 0.05, power: 0.8). (P5 Ln79-80)

6. About the position of the electrodes, authors should provide a reference for these electrode sites.

Ans: Thank you for reviewer’s reminder. The reference of placing these EMG electrodes was added. (P6 Ln104 & P7 Ln111)

7. About the justification of the scaption position, please refer to a study for the EMG level stated (10-15% MVC).

Ans: Thanks for raising the question. This information is added. (P7 Ln124-125, reference 24)

8. Ln 202-203: Please refer to systematic reviews and meta-analysis (e.g. 3,4) and it is important to be cautious to assume that chronic pain has the same effect as acute pain. A systematic review of chronic pain did not find any consistent pattern on TMS variables 5 conversely to experimental acute pain which reduces corticospinal excitability 3,4.

Ans: Thank you for the valuable suggestion. This information is added. (P13 Ln205-209)

9. Ln 231-232: Authors can discuss/described a bit more the findings of Tsao et al. that showed a difference in CoG position in the M1 map in low back pain compared to a painfree group 6,7.

Ans: Thank you for reviewer’s suggestion. We add this information in the discussion. (P15 Ln239-241)

- 1 Strutton, P. H., Theodorou, S., Catley, M., McGregor, A. H. & Davey, N. J. Corticospinal excitability in patients with chronic low back pain. J Spinal Disord Tech 18, 420-424, doi:00024720-200510000-00008 [pii] (2005).

- 2 Massé-Alarie, H., Beaulieu, L. D., Preuss, R. & Schneider, C. Corticomotor control of lumbar multifidus muscles is impaired in chronic low back pain: concurrent evidence from ultrasound imaging and double-pulse transcranial magnetic stimulation. Exp Brain Res 234, 1033-1045, doi:10.1007/s00221-015-4528-x (2016).

- 3 Burns, E., Chipchase, L. S. & Schabrun, S. M. Primary sensory and motor cortex function in response to acute muscle pain: A systematic review and meta-analysis. Eur J Pain, doi:10.1002/ejp.859 (2016).

- 4 Rohel, A. et al. The effect of experimental pain on the excitability of the corticospinal tract in humans: A systematic review and meta-analysis. Eur J Pain 25, 1209-1226, doi:10.1002/ejp.1746 (2021).

- 5 Parker, R. S., Lewis, G. N., Rice, D. A. & McNair, P. J. Is Motor Cortical Excitability Altered in People with Chronic Pain? A Systematic Review and Meta-Analysis. Brain Stimul 9, 488-500, doi:10.1016/j.brs.2016.03.020 (2016).

- 6 Tsao, H., Danneels, L. A. & Hodges, P. W. ISSLS prize winner: Smudging the motor brain in young adults with recurrent low back pain. Spine (Phila Pa 1976) 36, 1721-1727, doi:10.1097/BRS.0b013e31821c4267 (2011).

- 7 Tsao, H., Galea, M. P. & Hodges, P. W. Reorganization of the motor cortex is associated with postural control deficits in recurrent low back pain. Brain 131, 2161-2171, doi:10.1093/brain/awn154 (2008).

Reviewer #2

1. Thanks for addressing my comments. Authors have satisfactorily replied to my concern and I am happy to endorse the paper. I only have a minor aesthetic comment about the quality of figure 3 A & B. It would be nice to improve the quality for overall readership of the article.

Ans: Thank you for reviewer’s valuable comment. We further improved the quality of fig 3A & B as suggested.
