# Supplementary material for: Altered corticospinal excitability of scapular muscles in individuals with shoulder impingement syndrome
Source: PLoS One. 2022 May 16;17(5):e0268533. doi: 10.1371/journal.pone.0268533 (PMC9109916; doi:10.1371/journal.pone.0268533)
Supplement: Attachment — Submitted filename: Responses to reviewers R3_0325.docx [file pone.0268533.s004.docx]

Reviewer #1: General comments:

I thank the authors for considering my comments. I would urge authors to be cautious in the language used for data interpretation. Differences between groups coming from cross-sectional studies cannot be interpreted as “changes”, “reorganisation” or “modification”. Only a longitudinal study allows to conclude this. Authors should change these terms used throughout the manuscript. Also, I think the English should be revised before publication of the manuscript.

Answer: Thanks for the suggestions. The manuscript has been proof read by an English native speaker, and terms such as reorganization, modification, changes have been revised according to your suggestion throughout the manuscript (highlighted).

Specific comments:

Ln 32 : Considering this is a cross-sectional study, it is not possible to conclude in a “reorganisation” of the corticospinal system since it is not known if this is a predisposing factors or a modification which occurs because of pain. Authors should use a the term “different organisation” or “suggest a reorganisation”

Answer: Thanks for the suggestion. The sentence is revised as “participants with SIS demonstrated different reorganization of the corticospinal system…”. (Ln34-35)

Ln 279-280: I would be careful in the language used to interpret the findings. The sentence “The present study proved that neurophysiological changes existed in people with SIS, but there were some limitations of our methodologies.” should be modified to “ The present study suggest […]”.

Answer: Thanks for the suggestion. The sentence is revised as “ The present study suggested […]”. (Ln 280)

Ln 320: “suggest” instead of “supported”

Answer: Thanks for the suggestion. The sentence is revised accordingly. (Ln 310)
